# Supplementary material for: A collaborative data storage with incentive mechanism for blockchain-based IoV
Source: PeerJ Comput Sci. 2025 May 27;11:e2849. doi: 10.7717/peerj-cs.2849 (PMC12192716; doi:10.7717/peerj-cs.2849)
Supplement: Supplemental Information 3 [file peerj-cs-11-2849-s003.docx]

1. **Security Analysis**

This section outlines a comprehensive approach to enhancing the collaborative data storage process in blockchain-based Intelligent Internet of Vehicles (BIoV) by integrating an incentive mechanism, leveraging decentralization, ensuring data consistency, and utilizing smart contracts to foster security, efficiency, and reliability within the network.

(1) To Mitigate Selfish Nodes: To mitigate selfish node behavior, the study introduces an incentive mechanism within the collaborative data storage process among RSU nodes. The incentive function $I_{RSU}$ is designed to reward nodes based on their contribution to data storage and offloading. The rewards earned by RSU nodes, denoted as $I_{RSU}^{t}$ at time *t*, can be used to purchase cloud storage space, or for operational needs such as maintenance, where $I_{RSU}^{t}$ is a function of the number of data blocks stored $S_{RSU}^{t}$, data offloading $O_{RSU}^{t}$, and overall system contribution. This ensures nodes are motivated to act in the network’s best interest as $I_{RSU}^{t}=f(S_{RSU}^{t},O_{RSU}^{t})$, where $f(\cdot)$ is a predefined incentive function based on the system's parameters.

(2) Decentralized Strategy: The decentralized architecture of the system is formalized via the blockchain’s distributed consensus. The nodes in the RSU network do not rely on a single central entity, which reduces the risks of a single point of failure (SPOF). Mathematically, this can be expressed as the system's fault tolerance function $F_{SPOF}$, where the system remains operational as long as more than a certain threshold of nodes,$N_{th}$, are functioning correctly. This can be modeled as:

$F_{SPOF}=P(at least N_{th}\mathrm{total} N \mathrm{nodes} \mathrm{are} functional)$ (19)

Where the failure probability is computed using a binomial distribution as $P\left( \mathrm{failure} \right)=(\begin{matrix} N \\ N_{th} \end{matrix}){(p)}^{N_{th}}{(1-p)}^{{N-N}_{th}}$, where *p* is the probability of a node's failure. This reduces the risks of centralized vulnerabilities and ensures robust data storage across RSU nodes.

(3) Consistency Ensured: To maintain data consistency, any changes made to the data stored in an RSU’s local database must be propagated to other nodes. This can be formalized as a consistency propagation function $C_{RSU}$, where each update to the node *i*'s local storage triggers a consistency check across other RSU nodes $N_{RSU}$*.* This is mathematically represented as:

$C_{RSU}=\sum_{i=1}^{N_{RSU}} (\Pi_{update}(D_{i}))$ (20)

where $D_{i}$ represents the data block stored at node 𝑖, and $\Pi_{update}$ is an indicator function that triggers updates to other nodes if $D_{i}$ has changed. The immutability of the blockchain guarantees that any changes to data *D* require recalculating the hashes of all subsequent blocks:

$H_{i}=H_{i-1}\oplus H(D_{i})$ (21)

where $H(\cdot)$ denotes the cryptographic hash function, ensuring that any change to a data block $D_{i}$ alters its hash, thus making tampering computationally infeasible.

(4) Utilizing smart contracts: Our solution employs smart contracts to implement an incentive mechanism. These smart contracts operate autonomously, eliminating the need for intermediaries. Their code is transparent and accessible for public scrutiny, which enhances the verification process to ensure the security and integrity of the contracts. Openness and automation contribute to a more trustworthy and efficient system.

The incentive mechanism is implemented via smart contracts, which ensure the automated and transparent execution of the contract terms. The contract’s logic is formalized as a utility function $U(\cdot)$, where the incentives $I_{RSU}$ are allocated based on the contributions of the nodes, and these contracts are governed by the following conditions:

$U\left( I_{RSU} \right)=\sum_{i=1}^{N} f(S_{{RSU}_{i}},O_{{RSU}_{i}})$ (22)

where $S_{{RSU}_{i}}$ and $O_{{RSU}_{i}}$ represent the storage and offloading activities of each RSU node, and the summation ensures that all contributions are aggregated. The transparency and audibility of smart contracts are guaranteed by their open-source nature and public accessibility, ensuring the trustworthiness of the incentives. This is mathematically backed by contract verifiability via the equation $P\left( valid contract \right)=1$, meaning the probability of contract failure is negligible, provided the system's logic is correctly implemented.

**B. Formal Description and Analysis**

The contract states ***M**** is a quintuple,

$\boldsymbol{M}^{\boldsymbol{*}}=(Q,\Sigma,\sigma^{*},s^{*},F^{*})$ (23)

where $Q=\{q_{1}^{*},q_{2}^{*},\ldots,q_{m}^{*}\}$ represents the set of all states of the contract state machine. Each state, $q_{i}^{*}=q_{i}$ (*i*=1,$\ldots$,*m*), $q_{i}^{*}$ corresponds to a specific stage in the contract’s lifecycle and is included in all states of the contract participant.$\Sigma$ is the set of all input events that trigger state transitions. These events determine the flow of the contract’s execution.$\sigma^{*}$ is the state transition function, which maps the set of all possible input events to the set of states, formally written as $\sigma^{*}: Q\times\Sigma\to Q$. This function defines how the contract moves from one state to another based on the input events. $s^{*}$ is the initial state of the contract, where $s^{*}\in Q$. This state represents the starting point of the contract before any events have occurred. $F^{*}$ is the set of terminating states, where $F^{*}\subset Q$. Once the contract reaches one of these terminating states, the contract execution ends.

For clarity, let’s take the example of a Secure Remote Purchase Contract (https://solidity-cn.readthedocs.io/zh/develop/solidity-by-example.html), which is commonly used to demonstrate the use of smart contracts on the Ethereum network. The formal description of the states and transitions for such a contract is as follows:

$Q=\{contract created, contract locked, contract idle\}$ (24)

The contract created represents the state when the contract has been initialized. Contract locked is the state when the contract is locked and waiting for external inputs, such as confirmation of the transaction. Contract idle is the state when the contract is either waiting for further events or has completed its execution.

The set of input events, $\Sigma$, that trigger state transitions is given by:

$\Sigma=\{terminate transaction, confirm purchase,conf-irm receipt\}$ (25)

The terminate transaction ends the contract execution. Confirm purchase signifies the buyer's acknowledgment of the purchase. Confirm receipt indicates that the buyer has received the purchased goods. The state transition function $\sigma^{*}$governs how the contract moves between these states based on the input events. Specifically, the initial state $s^{*}$ is the contract created, and the contract can transition to a terminated state, $F^{*}$ once the transaction is complete.
